# Supplementary material for: Informing patterns of health and social care utilisation in Irish older people according to the Clinical Frailty Scale
Source: HRB Open Res. 2021 May 18;4:54. [Version 1] doi: 10.12688/hrbopenres.13301.1 (PMC8220351; doi:10.12688/hrbopenres.13301.1)
Supplement: Peer review history

# Peer review: Reviewer response for version 1

**DOI:** 10.21956/hrbopenres.14478.r29592

**Published:** 2021-06-22

**Authors:** Jordi Amblàs-Novellas

**Affiliations:** 1 Central Catalonia Chronicity Research Group (C3RG), Centre for Health and Social Care Research (CESS), University of Vic/Central University of Catalonia (UVIC-UCC), Vic, Spain

In this interesting manuscript, the authors conducted a prospective analysis using data from the TILDA database in order to assess the relationship between the CFS categories (obtained via CFS classification tree method) with patterns of health and social care utilization in older people in Ireland. On the other hand, the authors demonstrate the dynamic nature of CFS states over time using a multi-state Markov model.

**GENERAL COMMENTS**

 The reviewer wants to congratulate the authors for the results obtained, which are very relevant, and for the very rich and interesting discussion developed.

 In the opinion of the reviewer, this study provides 4 very relevant contributions:

1. As far as the reviewer knows, the CFS Decision Tree recently published by Theou et al. (doi: 10.1093/ageing/afab006) ^1^ is used for the first time in a large database, demonstrating its usefulness in retrospective data analysis.
2. A particularly relevant element of this study is the assessment of both the use of health and social services. Unfortunately, many times the studies only report metrics from the health field, thus limiting the global analysis of costs in these populations.
3. Beyond the clinical utility of the CFS, it places great emphasis on the usefulness of the results obtained for health and social policies (for example, the allocation of care resources in older people).
4. Finally, this article provides more evidence on the dynamic nature of frailty, emphasizing the need for periodic assessment of the degree of frailty of the population (the authors propose that CFS status should be reviewed at least every 2 years in individuals, or sooner when there is a change of individual circumstances).

**SPECIFIC COMMENTS:**

**1.    TITLE:** The title is explicit and clear

**2.    ABSTRACT:** It is easy to follow, it highlights the main results and it conforms to the rules of the journal. As a minor comment, it would be useful to specify the numerical category of the CFS, together with its description. For example: 6% 'very fit' (CFS 1), 36% 'fit' (CFS 2), 31% 'managing well' (CFS3), ... "

**3.    BACKGROUND:** The background gives enough information to contextualize the research question. It explains appropriately the study aims. The reviewer has only one minor comment related to Figure 1: Although the intention of the authors is to demonstrate the interrelation of the different dimensions, the fact is that the figure is not enough explanatory. Perhaps using different colours for each of the geriatric syndromes would be clearer. On the other hand, note that the concept of "multimorbidity" is repeated.

**4.    METHODS:**  The aims, the design of the study, the characteristics of participants and the description of materials are developed adequately, and the statistical analysis is appropriate. 

 It is especially interesting the use of the alluvial charts for the visualization of the longitudinal CFS transitions, as well as the use of the multistate Markov models to estimate transition probabilities for the CFS states from wave × to wave × + 1.

 A small limitation of the methodological approach is the source of data collection:

1. Usually, the most reliable source for obtaining clinical data is direct patient assessment. In this case, obtaining the CFS has been inferred retrospectively from a database previously designed for another purpose. It should also be noted that in the original study by Theou *et al.* (doi: 10.1093/ageing/afab006) ^1^ the CFS classification had been performing by professionals synchronously with the Comprehensive Geriatric Assessment.
2. On the other hand, usually, the most reliable source for obtaining data related to the use of resources are those coming from the information/billing systems. In this case, the uses of resources (number of times they visited a range of medical services over the previous 12 months) have been obtained through people self-declaration.

Although the authors comment on the first limitation, it might also be advisable to mention this second one.

**5.    RESULTS:**  The results are clear and follow a logical sequence. A large number of figures are provided, which greatly facilitate the understanding of the results.

 The reviewer only has 2 minor comments:

1. It is surprising that CFS classes 8 (very severely frail) and 9 (terminally ill) were not observed in TILDA. As mentioned by the authors, the results certainly cannot be extrapolated to the most vulnerable population of severely frail and terminally ill individuals.  However, given the fact that the sickest people are often those who require more care and make greater use of health and social services, the non-representation of these classes could represent a bias and should be taken into account as an overall limitation of the study. This also has implications related to health and social policies.
2. Figure 9: It is not clear to the reviewer if the results represented in this figure correspond to absolute values of resource use for each CFS category, or if they have been adjusted according to the prevalence of each CFS category. For example: Is the total spending on day centres, 7.1%, attributable to 0.7% of the total number of people with CFS7?

**6.    DISCUSSION:**  The discussion is clear and well-focused, although it lacks detail in the comparison of the results obtained with the evidence published so far. Many of the authors' claims could be contrasted with some of the many interesting publications that analyze:

1) the outcomes and use of resources (especially healthcare resources) in relation to the degree of frailty, also using the CFS.
  For example:
https://bmcgeriatr.biomedcentral.com/articles/10.1186/s12877-020-01801-7 ^2^
https://www.ncbi.nlm.nih.gov/pmc/articles/PMC7458601/ ^3^
https://pubmed.ncbi.nlm.nih.gov/28112778/ ^4^
https://bmcgeriatr.biomedcentral.com/articles/10.1186/s12877-020-01800-8 ^5^
2) The dynamic evolution of frailty over time, also using the CFS.
  For example:
https://academic.oup.com/ageing/article/48/4/547/5480913 ^6^
https://onlinelibrary.wiley.com/doi/abs/10.1111/ggi.13522 ^7^
https://academic.oup.com/ageing/article/49/6/974/5825993 ^8^
https://doi.org/10.1016/S2666-7568(20)30059-3 ^9^
https://doi.org/10.1186/s12889-021-10688-x ^10^

Regarding the limitations, certainly, the non-inclusion of people with dementia involves an important bias. Some of the limitations previously discussed by the reviewer could also be incorporated.

 The conclusions provide, in a clear way, an explanation of the importance and relevance of the study reported.

Is the work clearly and accurately presented and does it cite the current literature?

Yes

If applicable, is the statistical analysis and its interpretation appropriate?

Yes

Are all the source data underlying the results available to ensure full reproducibility?

Yes

Is the study design appropriate and is the work technically sound?

Yes

Are the conclusions drawn adequately supported by the results?

Yes

Are sufficient details of methods and analysis provided to allow replication by others?

Yes

Reviewer Expertise:

Frailty, complexity, end-of-life, integrated care

I confirm that I have read this submission and believe that I have an appropriate level of expertise to confirm that it is of an acceptable scientific standard.

## References

[1] : A classification tree to assist with routine scoring of the Clinical Frailty Scale. *Age Ageing* .2021; 10.1093/ageing/afab006 10.1093/ageing/afab006 33605412PMC7929455

[2] : A scoping review of the Clinical Frailty Scale. *BMC Geriatrics* .2020;20(1) : 10.1186/s12877-020-01801-7 10.1186/s12877-020-01801-7 33028215PMC7540438

[3] : Using the Clinical Frailty Scale in Allocating Scarce Health Care Resources. *Can Geriatr J* .2020;23(3) : 10.5770/cgj.23.463 210-215 10.5770/cgj.23.463 32904824PMC7458601

[4] : Use of Health Resources and Healthcare Costs associated with Frailty: The FRADEA Study. *J Nutr Health Aging* .2017;21(2) : 10.1007/s12603-016-0727-9 207-214 10.1007/s12603-016-0727-9 28112778PMC12879863

[5] : Frailty and healthcare utilisation across care settings among community-dwelling older adults in Singapore. *BMC Geriatrics* .2020;20(1) : 10.1186/s12877-020-01800-8 10.1186/s12877-020-01800-8 33023490PMC7542115

[6] : Fluctuations in frailty among older adults. *Age and Ageing* .2019;48(4) : 10.1093/ageing/afz040 547-552 10.1093/ageing/afz040 31028381

[7] : Frailty state transitions and associated factors in South Australian older adults. *Geriatr Gerontol Int* .2018;18(11) : 10.1111/ggi.13522 1549-1555 10.1111/ggi.13522 30221449

[8] : Transitions between frailty states in the very old: the influence of socioeconomic status and multi-morbidity in the Newcastle 85+ cohort study. *Age and Ageing* .2020;49(6) : 10.1093/ageing/afaa054 974-981 10.1093/ageing/afaa054 PMC758352432342980

[9] : Changes in the severity and lethality of age-related health deficit accumulation in the USA between 1999 and 2018: a population-based cohort study. *The Lancet Healthy Longevity* .2021;2(2) : 10.1016/S2666-7568(20)30059-3 e96-e104 10.1016/S2666-7568(20)30059-3 36098163

[10] : Frailty status changes are associated with healthcare utilization and subsequent mortality in the elderly population. *BMC Public Health* .2021;21(1) : 10.1186/s12889-021-10688-x 10.1186/s12889-021-10688-x 33794860PMC8017879

# Peer review: Reviewer response for version 1

**DOI:** 10.21956/hrbopenres.14478.r29515

**Published:** 2021-06-07

**Authors:** José Alberto Avila Funes

**Affiliations:** 1 Instituto Nacional de Ciencias Médicas y Nutrición Salvador Zubirán, Mexico City, Mexico; 2 Inserm U1219 Bordeaux Population Health Center. University of Bordeaux, Bordeaux, France

I have read with interest the manuscript titled “Informing patterns of health and social care utilization in Irish older people according to the Clinical Frailty Scale” by O’Halloran *et al* submitted for publication to the HRB Open Research.

 This interesting manuscript addresses a topic of great importance and relevance. In an aging population, the use of better indicators of vulnerability (beyond chronological age alone) may be the best strategy for decision-making in terms of health policies. In this way, the authors use a valid scale to identify the most frail older people to show that, indeed, those in highly vulnerable conditions are those who use more resources, both at hospital and community levels.

 On the other hand, the use of Markov models seems an ingenious strategy to demonstrate the dynamics of frailty, to show the probability of changing from one state to another and, most importantly, to clarify the potential for intervention on frailty syndrome to prevent people from suffering from the deleterious health outcomes associated with this geriatric syndrome.

 The methodology is clear, and the TILDA cohort is correctly explained even for those who are not familiar with it.

 The statistical plan is also consistent with the objectives of the study.

 The results are clear. However, at some point they can be redundant in the sense that they all point in the same direction (thankfully). In this way, it is not clear to me if that many figures are necessary to show the gradient between greater frailty and a higher need for any type of health service. It is up to the authors and the editor to decide if there is room for everything in the publication. However, it is necessary to underline that the graphs elegantly illustrate the gradient seen between a state of progressive frailty and the greater need to use different health resources.

 In my opinion, the discussion is consistent with the objectives of the study. The usefulness of the CFS and its potential for application at different levels are resumed, including for public health policies. Limits are also recognized. In particular, the impossibility of applying it to extremely frail people. This fact should be underlined, and caution is encouraged in the interpretation of the results. The conclusion is also strong with everything mentioned in the text.

 From my point of view, it is not very clear to me what wants to be presented in Figure 1. The superposition of the multiple determinants of frailty is clear, perhaps it should be pointed out more clearly in the text at the bottom of the figure.

 Correct the “type” of TILDA survey data, near the bottom of page 14.

 This is a very nice work from the Irish group that deserves to be disseminated and replicated to improve its external validity.

 Thanks for letting me read it and comment on it.

Is the work clearly and accurately presented and does it cite the current literature?

Yes

If applicable, is the statistical analysis and its interpretation appropriate?

Yes

Are all the source data underlying the results available to ensure full reproducibility?

Yes

Is the study design appropriate and is the work technically sound?

Yes

Are the conclusions drawn adequately supported by the results?

Yes

Are sufficient details of methods and analysis provided to allow replication by others?

Yes

Reviewer Expertise:

Frailty and ageing.

I confirm that I have read this submission and believe that I have an appropriate level of expertise to confirm that it is of an acceptable scientific standard.

# Response

**Published:** 2021-06-10

**Authors:** Aisling O'Halloran

**Affiliations:** Trinity College Dublin, Ireland

**Reviewer 1:**

**José Alberto Avila Funes, Instituto Nacional de Ciencias Médicas y Nutrición Salvador Zubirán, Mexico City, Mexico;  Inserm U1219 Bordeaux Population Health Center. University of Bordeaux, Bordeaux, France.**

 The authors would like to thank Prof Avila Funes for his thoughtful and generous review of our paper.

 Our responses to the specific points raised by the reviewer are as follows:

***'The results are clear. However, at some point they can be redundant in the sense that they all point in the same direction (thankfully). In this way, it is not clear to me if that many figures are necessary to show the gradient between greater frailty and a higher need for any type of health service. It is up to the authors and the editor to decide if there is room for everything in the publication. However, it is necessary to underline that the graphs elegantly illustrate the gradient seen between a state of progressive frailty and the greater need to use different health resources'.***

**Response: **The authors agree that there is a strong degree of repetition regarding the different medical, community and informal and formal health care services. As HRB Open Research is an online Open Research platform, there are no restrictions to the number of table and figure elements we may include in the paper. The authors have decided to keep all of the figures as they can provide useful data for other researchers and policy makers. In addition, as the reviewer points out, the graphs illustrate the progressive gradient between greater frailty and higher use across a broad range of different types of health and social services. This underlines the utility of the CFS as a potential tool to aid in the equitable allocation of health services resources in this study

***'From my point of view, it is not very clear to me what wants to be presented in Figure 1. The superposition of the multiple determinants of frailty is clear, perhaps it should be pointed out more clearly in the text at the bottom of the figure'.***

**Response: **We have clarified our description of Figure 1 as follows:

 Figure 1. Multiple overlapping geriatric syndromes (black) and the dimensions screened by the Clinical Frailty Scale (red).

***'Correct the “type” of TILDA survey data, near the bottom of page 14'.***

**Response: **This typo ‘TLDA’ has been corrected to ‘TILDA’ at the bottom of page 14 as requested.
